# A dosimetric analysis of a spine SBRT specific treatment planning system

**DOI:** 10.1002/acm2.12499

**Published:** 2018-11-20

**Authors:** Daniel L. Saenz, Richard Crownover, Sotirios Stathakis, Niko Papanikolaou

**Affiliations:** ^1^ University of Texas Health San Antonio Mays Cancer Center San Antonio TX USA

**Keywords:** Monte Carlo, spine, stereotactic body radiosurgery, treatment planning

## Abstract

**Purpose:**

The Brainlab Elements treatment planning system utilizes distinct modules for treatment planning specific to stereotactic treatment sites including single or multiple brain lesions as well as spine. This work investigates the hypothesis that an optimization tailored specifically to spine can in fact create dosimetrically superior plans to those created in more general use treatment planning systems (TPS).

**Methods:**

Ten spine patients at our institution were replanned in Brainlab Elements, Phillips Pinnacle^3^, and Elekta Monaco. The planning target volume (PTV) included the vertebral body (in either the thoracic or lumbar spine), pedicles, and transverse processes. In all plans, the target was prescribed 20 Gy to 95% of the PTV. Objectives for the study included D5%<25 Gy and spinal cord D0.035cc < 14 Gy. Plans were evaluated by the satisfaction of the objectives as well total monitor units (MU), gradient index (GI), conformity index (CI), and dose gradient (distance between 100% and 50% isodose lines) in a selected slice between the vertebral body and spinal cord.

**Results:**

All TPS produced clinically acceptable plans. The sharpest dose gradient was achieved with Elements (mean 3.3 ± 0.2 mm). This resulted in lowest spinal cord maximum point doses (6.6 ± 1.0 Gy). Gradient indices were also the smallest for Elements (3.6 ± 0.5). Further improvement in gradient index and spinal cord sparing were not performed due to the subsequent violation of the PTV D5% < 25 Gy constraint or the loss of conformity due to the loss of coverage at the PTV‐spinal canal interface.

**Conclusions:**

Brainlab Elements planning which relies on arc duplication to specifically optimize for spine anatomy did result in dosimetrically superior plans while holding prescription levels constant. While any planning system can improve upon specific dosimetric objectives, the simultaneous satisfaction of all constraints was best achieved with Brainlab Elements.

## 1 INTRODUCTION

Stereotactic body radiotherapy (SBRT) of spinal lesions has been increasingly utilized in radiotherapy for spine metastases as well as for primary tumors.^1^, ^2^ It also has a role in the retreatment setting.^3^ The increased use of this technique can be attributed to advances in localization accuracy both in terms of immobilization devices and precise image guidance. Studies estimate the localization accuracy of cone‐beam CT (CBCT), Cyberknife, and ExacTrac spine SBRT at submillimeter levels in each direction.^4^, ^5^, ^6^, ^7^ With an expanded role for this treatment modality, treatment planning, and delivery efficiency as well as the ability to optimize ideal dose distributions are critical.

Brainlab has recently released Elements, its most recent treatment planning approach for stereotactic applications. The package includes tools for Cranial SRS, Multiple Brain Mets SRS, Spine SBRT as well as contouring tools for cranial and spine applications. Within these Elements are tools for image fusion, the correction of spatial distortions and spine curvature in MR scans, and automatic contouring tools. The Elements are designed specifically for the region being treated. For example, when contouring a gross target volume (GTV) for a spinal lesion, the anatomical mapping will automatically generate a CTV contour which expands to encapsulate the spinal region to be included per International Spine Consortium Consensus Guidelines.^8^ In the dose optimization process, Brainlab Elements Spine SBRT will also enable arc splitting, a technique which creates additional arcs focusing on a specific segment of the planning target volume (PTV) in an effort to reduce complications due to concavities in the optimization. The intent is to create a rapid dose fall off between the target and spinal cord and other organs at risk (OAR) with clinically acceptable peaking doses and dose conformality. The aim of this study is to validate this tool by creating similar plans in other treatment planning systems (TPS) (Phillips Pinnacle^3^ and Elekta Monaco) and examine the dosimetric benefit.

## 2 MATERIALS AND METHODS

Ten (n = 10) patients previously treated at our institution were selected for this planning study based on identification of a single vertebral body with a GTV. The simulation CT scans alone were sent to Brainlab Elements where planning was first performed. In Elements, the CTV was manually generated to include the entire vertebral body, pedicles, and transverse processes. No additional margin was used for setup uncertainties to generate the PTV in this planning study. The mean PTV volume was 37.2 cc. The spinal cord was segmented through the vertebra of interest. Of the ten patients, seven patients had lesions in the thoracic spine while three were in the lumbar spine.

**Figure 1:**
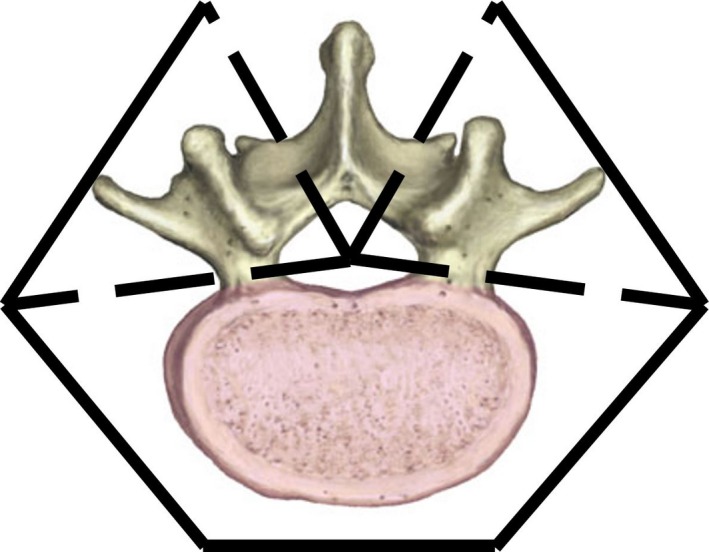
The division of the spine is shown for arc splitting in Elements. Dotted lines indicate the individual sectors treated with separate arcs in Elements. The divisions are places so as to minimize concavities in the target shape for an individual arc.

The clinical protocol set in Elements included covering 95% of the PTV with the prescription isodose line of 20 Gy with a D5% constraint of 25 Gy to control the hot spot. The prescription of 20 Gy was selected to push the optimization of the TPS sufficiently. This is higher than a more common prescription of 16 Gy (a review from Heron et al.^9^ showed a mean single fraction prescription dose of 16.3 Gy). The spinal cord constraints were D0.035cc < 14 Gy, D0.35cc < 10 Gy, D1.2cc < 7 Gy. A Monte Carlo capable beam model from a Novalis TX was used for treatment planning. VMAT beam geometry was also dictated in Elements, where an arc template was configured with two arcs (348 degree arc between IEC61217 gantry angle 185 and 173). Isocenter was placed in the centroid of the PTV. The collimator angle was set to 100 degrees. Elements arc duplication was enabled with the maximum number of arcs set at 6. This gives the planning system the ability to add additional arcs to treat particular sectors of the PTV independently. Elements divides the unique sectors among arcs by generating division lines which minimize target concavity. Figure 1 illustrates the effect. For this reason, Elements planning was performed first, so that the total number of arcs was known for the creation of similar beam geometries in other planning systems. A Monte Carlo dose calculation uncertainty of 2% per calculation was used, with a dose calculation grid spacing of 2 mm. Coverage as close to 95% as possible was pursued. The planning philosophy was to maximize the dose falloff outside the PTV and lower the spinal cord maximum dose as much as possible all while maintaining target coverage and keeping D5% < 25 Gy. Optimization was halted when conformity began to suffer due to excessive cord sparing or the hot spot climbed beyond 25 Gy due to constriction of the 50% isodose line. After dose was optimized in Elements, the CT and structures were exported to Philips Pinnacle^3^ and Elekta Monaco.

In Pinnacle^3^ and Monaco, identical beam geometry and calculation parameters were used for plans created in these systems. This included the same isocenter coordinate, the same gantry start and stop angles, the same collimator angles, and the same calculation uncertainty and grid spacing. Identical target coverage was used as well. In Pinnacle^3^, SmartArc was used, with a minimum segment area of 4 cm^2^, and minimum segment MU of 2. Final gantry spacing was set at 4 degrees (same as Elements). Adaptive Convolve was used for final dose calculation. In Monaco, the two arcs were specified with 180 maximum control points per arc. The maximum number of arcs was set to 2 (4 total) for those cases in which Elements used arc splitting, and at 1 (2 total) for the cases were Elements did not. In the end, identical numbers of arcs were used between all plans. Minimum segment width was set to 0.5 cm and segment shape optimization was used.

The same planning strategy was used in these planning systems as in Elements. That is to say, starting optimization objectives included 95% coverage of PTV (“min DVH” in Pinnacle^3^, “target penalty” in Monaco), D5% < 25 Gy (“max DVH” in Pinnacle^3^, “quadratic overdose” in Monaco), spinal cord maximum dose of 14 Gy (“max dose” in both Pinnacle^3^ and Monaco). Ring structures were employed in Pinnacle^3^ for GI control but not in Elements as this is optimized behind the scenes invisible to the user. In Monaco, normal tissue sparing was accomplished with a maximum dose cost function with a shrink margin (avoiding penalizing voxels within some specified distance from a target). Weightings between optimization objectives were set manually in Pinnacle^3^, set to “auto” in Monaco, and controlled with slider bars in the Elements interface. The manual process of optimization refinement was performed by pushing harder on normal tissue sparing and spinal cord sparing before target coverage and heterogeneity were compromised.

In addition to assessing the ability to meet planning objectives, plan evaluation was performed by recording the total monitor units (MU), gradient index (GI) (volume of 50% isodose volume relative to PTV volume), conformity index (CI) (volume of 100% isodose volume relative to PTV volume), and dose gradient (distance from the 100% to 50% isodose lines in the anterior aspect of the interface between the PTV and spinal cord in the isocenter slice).

## 3 RESULTS

**Figure 2:**
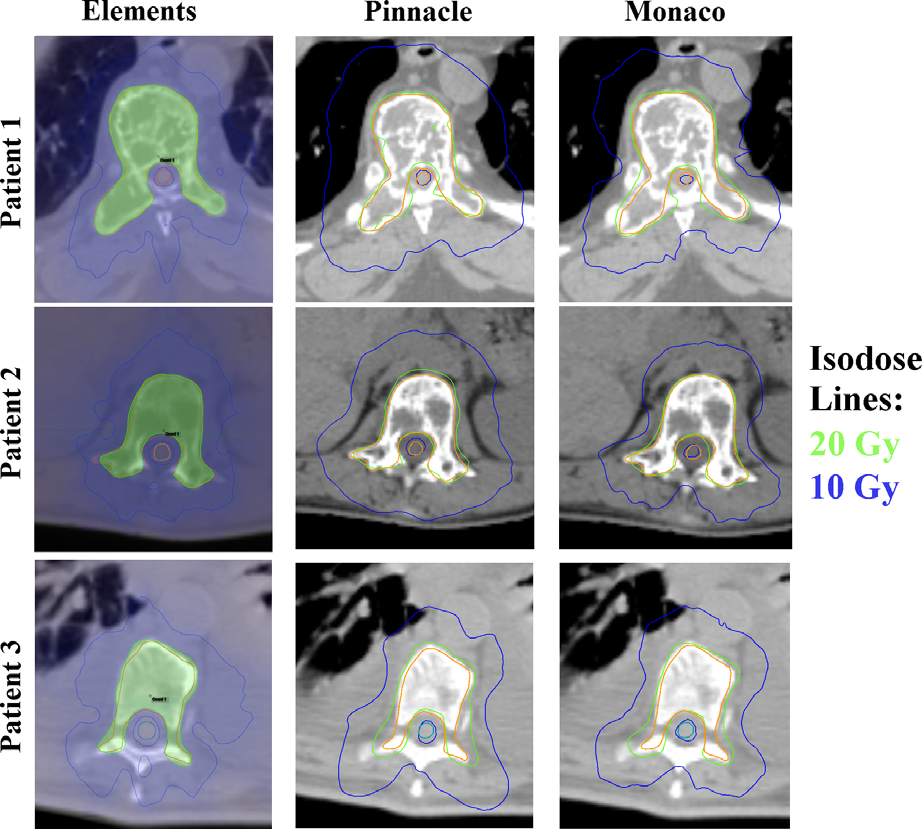
Sample isodose curves from the three treatment planning systems are shown for three patients. The PTV and spinal cord as well as the 20 and 10 Gy isodose lines are shown.

**Figure 3:**
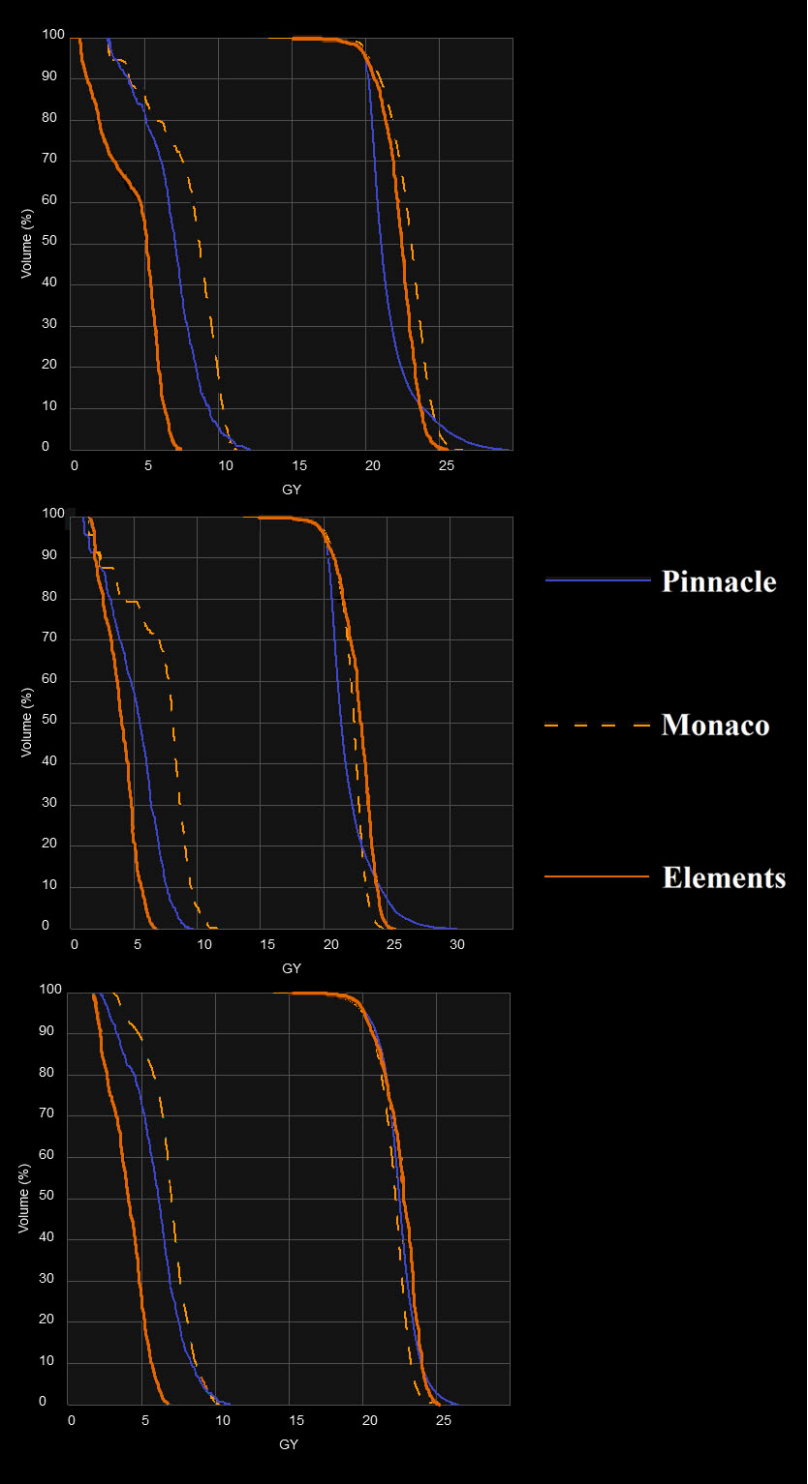
Sample DVH graphs from the three treatment planning systems for three patients. The PTV and spinal cord are plotted.

In Brainlab Elements, the optimization created two VMAT arcs for four patients and four arcs for six patients. Target coverage at 20 Gy was achieved at 95.8 ± 0.3% in Elements and at exactly 95.0% in the other two planning systems. Spinal cord maximum dose objectives were easily met in all planning systems, but with much lower maximum cord doses in Elements. Table 1 summarizes the dosimetric evaluation criteria. Figures 2 and 3 summarize sample dose volume histograms (DVH) and dose distributions in all three TPS.

**Table 1:** Summary of objectives and evaluation criteria of plans optimized in the three treatment planning systems. Values are averaged across the ten cases and the plus/minus numbers indicate standard deviations

	Elements	Pinnacle^3^	Monaco
Monitor units	7669 ± 1417	6836 ± 921	9177 ± 1189
PTV coverage (%)	95.8 ± 0.3	95.0 ± 0.1	95.0 ± 0.0
PTV D5% (Gy)	24.2 ± 0.3	24.5 ± 1.0	24.5 ± 0.7
Spinal cord maximum dose (Gy)	6.6 ± 1.0	10.4 ± 0.6	9.7 ± 1.1
Gradient index (GI)	3.6 ± 0.5	5.2 ± 0.7	4.5 ± 0.5
Conformity index (CI)	1.1 ± 0.02	1.2 ± 0.1	1.2 ± 0.04
Distance 20 Gy IDL to 10 Gy IDL (mm)	3.3 ± 0.2	3.9 ± 1.0	4.1 ± 0.4

In Elements, the spinal cord maximum dose and gradient index were lowered as much as possible while keeping the maximum dose in the PTV within tolerance and while keeping the CI near 1.1. In Pinnacle^3^ and Monaco, further lowering of spinal cord dose and dose gradient was prevented by the D5% nearing 25 Gy and conformity nearing 1.2, as can be seen in Table 1. As these evaluation criteria were nearing the edge of clinically acceptable plans, further optimization was halted. In the final analysis, both spinal cord maximum dose and gradient indices were significantly lower in Elements than in the other planning systems. Furthermore, the dose gradient (20 to 10 Gy isodose lines) was 0.6 mm sharper than Pinnacle^3^ and 0.8 mm sharper than in Monaco. Target coverage was mostly equal, while more monitor units were required in Monaco, but less In Pinnacle^3^.

A two‐tailed Wilcoxon signed‐rank test (due to the low sample size) was also conducted to evaluate the null hypothesis of no difference between the TPS. No statistical difference was found for CI for Elements plans vs either Pinnacle^3^ or Monaco. For GI, the W‐values were 0 for Elements plans vs both Pinnacle^3^ and Monaco, respectively, which is less than the critical value of 8 meaning the improvements in gradient index were statistically significant with Elements. Spinal cord maximum doses were also statistically different with W‐values of 0. W‐values of 5 and 0 were found for the distances from 100% to 50% isodose lines for Pinnacle and Monaco respectively.

## 4 DISCUSSION

The main dosimetric findings include the lower GI and spinal cord maximum dose with similar conformity and dose heterogeneity. These results were compared with a study in the literature investigating spinal radiosurgery plans across systems and across institutions.^10^ In that study, with 95% PTV coverage, the average CI was 1.47 and ranged from 1.08 to 2.04. CI in this study is clearly on the lower end of this range. The ability to better spare normal tissues such as the spinal cord is made possible by the arc splitting concept employed by Elements. The benefit is not only due to the second pass to create more control points to meet objectives, but rather due to treating distinct sectors of the target in separate arcs particularly in regions of target concavity (such as the spinal cord). At gantry angles of 90 and 270, the optimizer is not forced to put fluence through the spinal cord to irradiate the bilateral transverse processes. If it is only asked to treat the proximal transverse process, the optimizer does not face as much conflicting penalty from cord dose overirradiation and distal transverse process underirradiation. A separate pass can focus on the contralateral side.

The physical dose gradient from 20 to 10 Gy isodose line was smaller in Elements, but it is difficult to determine if this is a conclusive finding since the differences were relatively modest. In patients not requiring as extensive treatment of the transverse processes, it is expected that the advantages of Elements would be smaller.

Based on the excellent spinal cord sparing, it may be possible to investigate dose escalation for such spine SBRT cases. In fact, Moussazadeh et al.^11^ have reported use of 24 Gy for SBRT with mean PTV volumes of 67.9 cc, approximately twice the PTV volume in this study. In that study, overall maximum dose to the spinal cord was 13.4 Gy. With a mean spinal cord maximum doses of 6.6 Gy in the Elements plans in this study, escalation to 24 Gy would be possible with even lower risks of spinal cord toxicities than in that study.

Calculation time is often a significant parameter for consideration of the efficiency of the treatment planning process, particular for stereotactic radiotherapy given the importance of the temporal gap between the MR study, CT simulation, and treatment. Calculation times are difficult to compare between planning systems given variations in computer hardware capabilities and calculation volumes among other complicating factors. Nevertheless, typical pencil‐beam algorithm optimization times were on the order of a minute in Brainlab Elements, extending to several minutes for Monte Carlo optimization. Final planning times in Brainlab Elements after alteration of a several planning tools resulting in plan creation in 30–45 min.

Brainlab Elements is also characterized by a protocol‐driven approach to treatment planning. Prescriptions, constraints to OAR, and relative importance of organs are set offline in protocols, moving much of the plan modification behind the scenes. Such a workflow moves into the automated treatment planning regime, displacing much of the time spent entering objectives and technical parameters from patient‐specific planning to the initial commissioning of the software. Careful attention must be paid upfront, however, to the input of the physician and physicist so that clinical prescriptions and delivery approach can be settled before final commissioning of the treatment technique is performed.

## 5 CONCLUSIONS

The spine module in Brainlab Elements was evaluated from a treatment planning standpoint on its ability to optimize spine SBRT dose distributions. Similar plans were generated in other commonly used TPS to determine if the anatomy‐specific Elements plans are dosimetrically superior to others. Specifically, the spinal cord maximum dose, gradient index, and steepness of the dose falloff between the PTV and spinal cord were found to be improved in Elements plans.

## CONFLICTS OF INTEREST

This work was supported in part by a grant from Brainlab.
